# Excess costs of dementia disorders and the role of age and gender - an analysis of German health and long-term care insurance claims data

**DOI:** 10.1186/1472-6963-12-165

**Published:** 2012-06-19

**Authors:** Larissa Schwarzkopf, Petra Menn, Reiner Leidl, Sonja Wunder, Hilmar Mehlig, Peter Marx, Elmar Graessel, Rolf Holle

**Affiliations:** 1 Helmholtz Zentrum München, Institute of Health Economics and Health Care Management, Ingolstaedter Landstrasse 1, 85764, Neuherberg, Germany; 2 AOK Bavaria - Health Insurer, Stromerstrasse 5, 90443, Nuremberg, Germany; 3 Eisai GmbH, Lyoner Strasse 36, 60528, Frankfurt, Germany; 4 Pfizer Deutschland GmbH, Linkstrasse 10, 10785, Berlin, Germany; 5 University Hospital Erlangen - Clinic for Psychiatry and Psychotherapy, Schwabachanlage 6, 91054, Erlangen, Germany

**Keywords:** Payer perspective, Administrative data, Net expenditures, Economics, Health care sector, Long-term care sector, Nursing care, Case–control study

## Abstract

**Background:**

Demographic ageing is associated with an increasing number of dementia patients, who reportedly incur higher costs of care than individuals without dementia. Regarding Germany, evidence on these excess costs is scarce. Adopting a payer perspective, our study aimed to quantify the additional yearly expenditures per dementia patient for various health and long-term care services. Additionally, we sought to identify gender-specific cost patterns and to describe age-dependent cost profiles.

**Methods:**

The analyses used 2006 claims data from the AOK Bavaria Statutory Health Insurance fund of 9,147 dementia patients and 29,741 age- and gender-matched control subjects. Cost predictions based on two-part regression models adjusted for age and gender and excess costs of dementia care refer to the difference in model-estimated means between both groups. Corresponding analyses were performed stratified for gender. Finally, a potentially non-linear association between age and costs was investigated within a generalized additive model.

**Results:**

Yearly spending within the social security system was circa €12,300 per dementia patient and circa €4,000 per non-demented control subject. About two-thirds of the additional expenditure for dementia patients occurred in the long-term care sector. Within our study sample, male and female dementia patients incurred comparable total costs. However, women accounted for significantly lower health and significantly higher long-term care expenditures. Long-term care spending increased in older age, whereupon health care spending decreased. Thus, at more advanced ages, women incurred greater costs than men of the same age.

**Conclusions:**

Dementia poses a substantial additional burden to the German social security system, with the long-term care sector being more seriously challenged than the health care sector. Our results suggest that female dementia patients need to be seen as a key target group for health services research in an ageing society. It seems clear that strategies enabling community-based care for this vulnerable population might contribute to lowering the financial burden caused by dementia. This would allow for the sustaining of comprehensive dementia care within the social security system.

## Background

Dementia disorders are characterized by a progressive loss of cognitive function accompanied by increasing need for support in daily life. So far, dementia is incurable, and pharmacological and non-pharmacological interventions can only delay its progression [1].

Regarding Germany, about 7.2% of the resident population aged 65 and older is estimated to suffer from dementia [2,3]. The risk of developing dementia rises in older age [2,3], and thus the number of patients is expected to double to 2.4 million as a result of demographic ageing by 2050 [4].

About 85% of Germany’s inhabitants are insured within the social security system, which is characterized by pay-as-you-go financing, income-dependent but not risk-dependent insurance contributions and the availability of long-range health and long-term care services for a small copayment [5]. In an ageing society, these principles are associated with a widening gap between receipts and expenditures. Hence, the interest in quantifying the economic burden of age-associated diseases as dementia grows.

Within the last 15 years, several studies have estimated annual per capita expenditures of Statutory Health Insurance (hereafter SHI) and Compulsory Long-Term Care Insurance (hereafter LTCI) on patients with Alzheimer’s dementia. To ensure comparability with our own data referring to 2006 as the base year, we first converted the reported figures to Euros by applying the average currency exchange rate of the corresponding year and, secondly, inflated these figures to 2006 values by applying the German gross domestic product deflator.

Based on expert interviews, Hallauer and colleagues calculated circa €15,000 (original value: DM27,500) as the annual cost of care [6]. By means of patient records, Schulenburg and collaborators estimated a minimum of €6,600 if all patients had received community-based care from relatives and a maximum of €12,400 if all patients had received institutional care (original values: $8,100 and $15,200) [7]. Kiencke et al. presented a claims data-based Markov model, indicating costs of circa €13,400 per year survived across different pharmacological treatment groups (original value: €13,100) [8].

Disregarding a comparison with non-demented control subjects, the cited research left unclear whether dementia per se is linked to an increase in health and long-term care expenditures.

International studies suggest that dementia patients incur far higher costs than individuals without dementia irrespective of the perspective adopted [9-13]. In the German context, information on these so called excess costs is scarce. We found one study adopting a societal perspective [14] and one piece of research adopting the perspective of the social security system [15].

Standardized for the German resident population aged 60 years and older, the latter estimated annual per capita expenditures of circa €13,300 (original value: €13,800) for insurants with a dementia diagnosis compared with circa €3,600 (original value: €3,700) for insurants without such a diagnosis [15]. Around 63% of additional costs were attributable to LTCI.

Further evidence is needed regarding the contribution of different health and long-term care services to the increased spending. Using claims data from a large regional SHI fund for a case–control comparison, this paper has the threefold objective of:

(1) comparing expenditures for dementia patients and non-demented control subjects within the distinct service categories of SHI and LTCI;

(2) searching for potential gender differences regarding total costs of care and costs within the distinct service categories; and

(3) analysing age-dependent cost profiles.

## Methods

### Data source and sample selection

AOK Bavaria is the leading SHI fund in the district of Middle Franconia covering about 50% of the resident population aged 65 years and older. For 2005–2007, we received their complete claims data for all insurants in this age group. Data provision was carried out according to German data protection laws, and AOK Bavaria approved the use of the data set for the intended analyses.

We restricted our selection process to insurants with records in all three years born before 1941, leading to a baseline data set of 151,171 individuals. The allocation to the case and the control group was based on 2005 and 2006 data, and costs were evaluated for 2006.

We identified people suffering from dementia by constructing ‘dementia quarters’, based on inpatient diagnoses, outpatient diagnoses and anti-dementia drug prescriptions. Dementia diagnoses included the ICD-10 codes F00, F01, F02, F03 and G30. Anti-dementia drug prescriptions consisted of donepezil, galantamine, rivastigmine (all ATC code ‘N06DA’) and memantine (ATC code ‘N06DX01’). In Germany, cholinesterase inhibitors and memantine are licensed for the treatment of Alzheimer’s dementia only; thus, the prescription of a corresponding drug was seen as on a par with an explicit diagnosis. Whenever one of these ICD-10 codes and/or one of these ATC codes was documented at least once within a quarter, the corresponding quarter was defined as a dementia quarter.

A total of 14,721 insurants showed dementia quarters. To increase the validity of diagnoses, we excluded 2,136 individuals with their first documented dementia quarter in IV/2006 and 545 individuals not continuously enrolled in AOK Bavaria. By this method, 12,040 dementia patients remained.

Considering the chronic course of dementia, we required the documentation of at least three dementia quarters within four consecutive quarters [16,17] to classify an insurant as an ‘assured dementia case’. At least one of these dementia quarters had to be in 2006, the year of cost analysis. To avoid loss of information, we defined all patients with dementia quarters who did not fulfil these two requirements as ‘potential dementia cases’. This process led to 9,147 patients with assured dementia (case group) and 2,893 patients with potential dementia. The latter were included in a sensitivity analysis.

From the remaining subjects without dementia quarters (n = 136,450), we dropped 1,122 individuals with piracetam prescriptions. Treatment with piracetam is common in dementia disorders, but it is not an exclusively dementia-specific medication. We could not control for the consumption of ginkgo biloba because SHI reimbursement is limited for these pharmaceuticals, and therefore they are usually paid out-of-pocket.

Age- and gender-matched control subjects to the assured dementia patients were randomly selected from the 135,328 potential control subjects in a 4:1 ratio. As the dementia prevalence in men aged 84 onwards and in women aged 82 onwards exceeded 20%, an exact 4:1 matching was not feasible. For these age groups, we included all remaining potential control subjects, and the eventual control group contained 29,741 individuals.

The inclusion criteria for our study sample are summarized below:

(1) Aged at least 65 years on 1 January 2006.

(2) Continuously insured with AOK Bavaria in 2005 and 2006.

(3) Case group

(a) At least three dementia quarters within four consecutive quarters of 2005 and 2006

(b) At least one dementia quarter in 2006

(4) Control group

(a) No dementia quarter in 2005 and 2006

(b) No piracetam prescription in 2005 and 2006

### Costs of care

The German social security system provides health insurance, long-term care insurance, pension insurance, (occupational) accident insurance and unemployment insurance. Payments of the last two benefits are dispensed with on retirement, and the amount of retirement pay is not affected by health status. Thus, cost differences in the health and long-term care sector are an acceptable base on which to estimate excess expenditures for dementia patients within the social security system [15].

#### Background information on SHI

SHI covers hospital care, ambulatory treatment by general practitioners and medical specialists, medication (except for drugs paid out-of-pocket), medically indicated non-physician services and medical aids, home health care and rehabilitation. SHI spending equals the sum of 2006 expenditures for these services.

Whenever a treatment took place only partly in 2006, we calculated treatment duration within the observation period. Then, costs were attributed proportionally to the time of resource use, implying constant amounts per day.

Physician services are billed via a fee for service system that settles a specific score for each service. The monetary value of services is calculated by multiplying this score by a quarter- and specialization-specific point value. We converted the scores into Euro amounts by applying the corresponding monetary point values published by the Association of SHI Physicians of Bavaria.

The medication category includes all prescribed drugs. The corresponding pharmacy retail prices are available from the Scientific Institute of the AOK. However, pharmacies distribute not only drugs but also to some extent medical aids (e.g. blood pressure meters, blood glucose meters or bandages), which also occur under the heading medication because of accounting rules.

Hospitals provide inpatient treatment, emergency care and outpatient services (e.g. day surgery, preadmission services, post-discharge services), which are for the most part reimbursed via a diagnosis-specific fixed remuneration additionally weighted by the patient’s comorbidity status (Diagnosis Related Group, DRG). Overall spending is explicitly documented in the claims data.

The expenditures for non-physician services and home health care reported in the claims data refer to prescriptions. A prescription for non-physician services covers a fixed number of treatment sessions, whereas home health care is limited to a distinct time horizon. The absolute payment amount per prescription depends first on the number of contacts between patient and provider and second on the type of services provided. These details on intensity of resource utilization are not traceable; instead, only the total sum accrued for the delivered services is reported.

#### Background information on LTCI

Despite being designed as an individual social insurance branch, LTCI is managed under one umbrella with SHI.

It addresses people with continuous need for support due to physical impairment and supports various community-based and institutional long-term care services.

The Code of Social Law defines three care levels, each connected to a fixed monthly tariff for community-living or institutionalized beneficiaries. These payments represent supplementary financial support and are not intended to provide full coverage of costs incurred for long-term care. In order to profit from LTCI services, an application is required followed by an appraisal from the Medical Review Board of the SHI Funds.

To quantify per capita costs within the social security system, we added LTCI and SHI expenditures and labelled the sum costs of formal care.

### Statistical analysis

For a first impression, we compared unadjusted mean per capita expenditures for dementia patients and non-demented control subjects using Wilcoxon tests, which account for the highly skewed distribution of cost data.

Our primary analysis adjusted for the covariates age and gender. It applied generalized linear models (hereafter GLM) assuming a gamma distribution with log link. Cost differences were evaluated via two different approaches: approach 1 for categories in which costs were incurred by almost every patient and approach 2 for categories with a user quota below 90%.

In approach 1, a one-step GLM adjusted for age and gender was performed to estimate annual per capita expenditures for cases and control subjects. We assigned a small positive value to the few individuals without costs to avoid their exclusion from the analyses. Approach 1 was used for the domains general practitioner, medication, SHI expenditures and costs of formal care.

For the categories medical specialist, hospital treatment, non-physician services, medical aids, home health care, rehabilitation and long-term care services, we followed approach 2, in which we calculated two-part regression models [18]. Part 1 estimated the probability of positive expenditures based on logistic regression. In part 2, a gamma model, as described above, was applied to estimate the annual amount of expenditures for those with positive expenditures. Both stages included age and gender as covariates. To derive per capita costs, the estimated probabilities for positive expenditures were multiplied by the predicted costs per user. Recycled predictions with dementia as the coefficient of interest were used to investigate differences between the case and the control group combined for both stages [19]. In this approach, two predictions are derived based on the regression estimates: first, assuming all subjects do not have dementia; second, assuming that all have dementia. The mean differences between these predictions are the excess costs of dementia adjusted for covariates [20].

Adjusted SHI expenditures and adjusted costs of formal care as per the regression model differed slightly from the sum of adjusted mean costs in the distinct categories, which results from the stepwise calculation within the two-part model (approach 2). Moreover, the two-part model provided two p-values, the first referring to differences in the probability of service use, and the second referring to cost differences among service users. We assumed a significant difference at the patient level if both parts pointed in the same direction and if at least one of the two p-values was significant.

We also tested an extended model which considered all possible single interaction terms between gender, age and dementia. Cost estimations were comparable, but the interpretation of p-values would have been less straightforward. Thus, we chose the simple model without interaction terms.

Excess costs of dementia care were obtained by subtracting the estimated costs per control subject from the estimated costs per case [21]. For total costs, we calculated the 95% confidence interval of adjusted costs based on 1,000 nonparametric bootstrap replications.

Supplementary to the primary analysis, we performed two sensitivity analyses. Sensitivity analysis 1 defined dementia less strictly and included the 2,893 potential cases. In sensitivity analysis 2, the regression models considered dummy variables for specific comorbid conditions and death in 2007. We included the five most common comorbidities from the complex ‘cardiovascular and metabolic disorders’ [22], which were hypertension (ICD I10–I15), lipid metabolism disorder (ICD E78), diabetes mellitus (ICD E10–E14), chronic ischaemic heart disease (ICD I20, I21, I25) and cardiac insufficiency (ICD I50). An insurant was considered to suffer from the respective disease if a corresponding ICD was coded at least once in 2006.

As a secondary analysis, we investigated gender differences regarding costs of care, within a stratified model. Again, recycled predictions were calculated, this time with gender as the coefficient of interest. Here, the interaction term ‘dementia*gender’ was also included in the previously described GLMs. Further interactions were disregarded, which enables an isolated observation of the gender effect in the case and in the control group.

Within a tertiary analysis, we evaluated a possible non-linear association between age and costs of formal care, SHI expenditures and LTCI expenditures. We therefore fitted a generalized additive model allowing a separate smooth function of age for the four subgroups of male control subjects, female control subjects, male cases and female cases. The smooth functions were estimated using thin plate regression splines, and smoothing parameters were estimated using generalized cross-validation [23].

A significance level of 5% was used for all analyses, which were performed with the software package SAS, version 9.2.

## Results

### Baseline characteristics

According to Table 1, dementia patients were around 2 years older than control subjects and had a higher share of females because of the imperfect matching.

**Table 1:** Baseline characteristics of the study sample

**Characteristics of the study sample**	**Dementia patients**	**Non-demented control subjects**	p-value*	*Potential dementia cases*
N	9,147	29,741		*2,893*
Mean age	81.6 (7.4)	79.6 (6.4)	<0.0001	*79.0 (7.7)*
Females (as a %)	6,819 (74.6%)	20,932 (70.4%)	<0.0001	*2,020 (69.8%)*
Beneficiary of long-term care (as a %)	6,037 (66.0%)	3,829 (12.9%)	<0.0001	*1,078 (37.3%)*
Community-living at 1 January 2006 (as a %)	6,212 (67.9%)	29,028 (97.7%)	<0.0001	*2,484 (85.9%)*
Shift to nursing home within 2006 (as a %)	654 (7.1%)	292 (1.0%)	<0.0001	*107 (3.7%)*
Death in 2007 (as a %)	1,621 (17.7%)	1,764(5.9%)	<0.0001	*311 (10.8%)*
Hypertension	5,768 (63.1%)	19,109 (64.3%)	0.04	*1,881 (65.0%)*
Lipid metabolism disorder	2,718 (29.7%)	10,825 (36.4%)	<0.0001	*967 (33.4%)*
Diabetes mellitus	3,446 (37.7%)	8,977 (30.2%)	<0.0001	*1,014 (35.1%)*
Ischaemic heart disease	2,747 (30.0%)	8,027 (27.0%)	<0.0037	*836 (28.9%)*
Congestive heart failure	3,075 (33.6%)	6,478 (21.8%)	<0.0001	*750 (25.9%)*

Data are mean (standard deviation) unless noted otherwise.

* Statistical significance at 5% level based on Chi^2^ tests for categorical variables and Kruskal–Wallis tests for continuous variables.

~ Statistical significance at 5% level adjusted for age and gender within logistic regression models.

After adjustment for these demographic differences, they still depended to a larger percentage on professional long-term care and were more likely to die within the year following the observation.

The potential dementia patients who were disregarded within the matching algorithm were younger and had a lower female quota than the cases and control subjects. Regarding the other baseline characteristics, they ranged between both groups with a slight tendency towards the case group.

Diabetes and heart diseases were more prominent in dementia patients but hypertension and lipid metabolism disorder in control subjects.

### Expenditures within the case and the control group

#### Costs of care and excess expenditures

**Figure 1:**
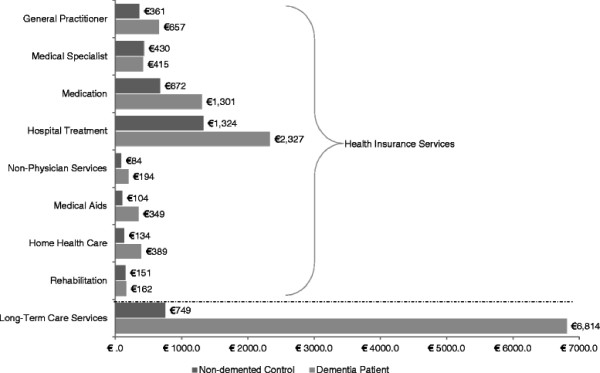
Unadjusted mean expenditures per year for dementia patients and non-demented control subjects.

The comparison of unadjusted means yielded annual expenditures of circa €12,600 for dementia patients and circa €4,000 for non-demented control subjects. As highlighted in Figure 1, costs for dementia patients were increased for all categories except medical specialists. Apart from rehabilitation, all observed differences were significant.

Table 2 summarizes the model-based cost estimates adjusting for age and gender differences in both groups. In this primary analysis, annual costs of formal care were circa €12,300 in the case group and almost equally distributed between SHI and LTCI. In the control group, the corresponding ratio was around 80:20, totalling annual expenditures of circa €4,000.

**Table 2:** Annual per capita costs (€) and estimated excess costs (€) adjusted for age and gender

	**Service component**	**Cost per dementia patient (n = 9,147)**	**Cost per non-demented control subject (n = 29,741)**	Excess expenditures**
**Approach 1 one-step model**	Costs of formal care * [95%-CI]	**12,343 [12,126; 12,572]**	**4,034 [3,957; 4,109]**	**8,309 [8,081; 8,552]**
	Health insurance expenditures*	***5,813***	***3,256***	***2,557***
	Medication	1,312	671	641
	*including anti-dementia drugs (two-step)*	*119*	*0*	*119*
	General practitioner	641	367	274
**Approach 2 two-step-model **	Medical specialist	432	429	3
	Hospital treatment	2,237	1,325	912
	Non-physician services	199	83	116
	Medical aids	339	106	233
	Home health care	361	138	223
	Rehabilitation	164	150	14
	***Long-term care services***	***6,353***	***797***	***5,556***

Data are means based on recycled predictions with dementia as the coefficient of interest.

The 95% confidence interval is based on 1,000 nonparametric bootstrap replications.

* Results of model estimation; the addition of mean costs per category yields slightly different figures.

** Defined as the difference of estimated means within both subgroups.

Focusing on the distinct categories itemised in Table 2, dementia patients incurred consistently higher spending than control subjects. The difference was not significant in the use of medical specialists. Total SHI expenditures were increased by circa 80% and LTCI expenditures by circa 700%. Around two-thirds of the excess expenditures on formal care were attributable to LTCI.

Table 3 illustrates how differences in per capita expenditures came about: first, except for rehabilitation, the probability of service use was significantly increased in dementia patients (p1). Second, the average spending on service users with dementia was in general remarkably higher than the average spending on service users without dementia (p2). Regarding medical specialists, the probability of service utilization and costs per user pointed in opposite directions. This resulted in an insignificant difference at the per capita level.

**Table 3:** Probability of service use and annual costs per user adjusted for age and gender

	**Service component**	**Dementia patients (n = 9,147)**		**Non-demented control subjects (n = 29.741)**		**p-values**	
		**Users (as a %)**	**Cost per user (in €)**	**Users (as a %)**	**Cost per user (in €)**	**p 1 probability of service use**	**p 2 cost per user**
**Approach 1 one-step model**	Costs of formal care*	**100.0**	**12,343**	**96.5**	**4,034**	**/**	**<0.0001**
	Health insurance expenditures*	***100.0***	***5,813***	***96.4***	***3,256***	**/**	**<0.0001**
	Medication	99.5	1,312	93.5	671	/	<0.0001
	* including anti-dementia drugs (two-step)*	*15.2*	*764*	*0.0*	*0*		
	General practitioner	99.4	641	93.7	367	/	<0.0001
**Approach 2 two-part model**	Medical specialist	81.4	517	81.0	524	0.0009	0.47
	Hospital treatment	42.5	5,504	28.2	4,697	<0.0001	<0.0001
	Non-physician services	27.0	720	21.6	390	<0.0001	<0.0001
	Medical aids	65.2	538	35.6	293	<0.0001	<0.0001
	Home health care	17.0	2,254	6.3	2,118	<0.0001	0.14
	Rehabilitation	4.8	3,416	4.8	3,135	0.9	0.0036
	***Long-term care services***	***66.0***	***9,918***	***12.9***	***5,483***	**<0.0001**	**<0.0001**

Data derive from one-step and two-step Generalized Linear Models with dementia as the coefficient of interest.

Significance at the patient level is estimated based on p1 and p2 in the two-part models and based on p2 in the one-step models.

p1 derives from the logistic model (approach 2, step 1) and p2 derives from the gamma model (approach 2, step 2 and approach 1).

* Results of model estimation; the addition of mean costs per category yields slightly different figures.

#### Sensitivity analyses

Including potential dementia cases in sensitivity analysis 1 only affected cost estimates within the case group. Compared with the primary analysis, SHI expenditures decreased by circa €200 and LTCI expenditures by circa €700. Altogether, annual costs of formal care were estimated as circa €11,400 (primary analysis: circa €12,300).

Sensitivity analysis 2, which adjusted for comorbidity, again revealed annual costs of formal care of circa €12,300 in dementia patients and circa €4,100 (primary analysis: circa €4,000) in non-demented control subjects. The excess expenditures for dementia patients were comparable to the primary analysis.

### Gender-specific cost profiles

#### Costs of care and excess expenditures for dementia patients according to gender

Similar to the primary analysis, the gender-stratified GLM revealed significant cost differences between male cases and male control subjects as well as between female cases and female control subjects regarding all service categories except medical specialists (results not shown).

Adjusted for age, annual costs of formal care amounted to circa €12,600 in male dementia patients and circa €4,300 in male control subjects. The corresponding figures observed in females were slightly lower with circa €12,200 in dementia patients and circa €3,900 in non-demented control subjects.

Compared with control subjects, male dementia patients incurred circa €5,300 extra in LTCI (circa €5,900 vs. circa €600) and circa €2,900 extra in SHI (circa €6,500 vs. circa €3,600). The excess expenditures for female dementia patients compared with female control subjects amounted to circa €5,600 in LTCI (circa €6,500 vs. circa €900) and circa €2,500 in SHI (circa €5,600 vs. circa €3,100).

#### Gender-specific cost profiles

The gender-specific cost compilation within the case group is described in Table 4. Male dementia patients incurred significantly higher SHI spending (circa €6,500 vs. circa €5,600), and female dementia patients incurred significantly higher LTCI spending (circa €6,500 vs. circa €5,900). The effects in the opposite direction led to comparable total costs of formal care (p = 0.17).

**Table 4:** Gender-specific annual mean expenditures within the case group adjusted for age

	**Service component**	**Male dementia patients (n = 2,328)**			**Female dementia patients (n = 6,819)**			**p-values**	
		**Users (as a %)**	**Cost per user (in €)**	**Cost per patient (in €)**	**Users (as a %)**	**Cost per user (in €)**	**Cost per patient (in €)**	**Probability of service use**	**Cost per user**
**Approach 1 one-step model**	Costs of formal care*	**100.0**	**12,648**	**12,648**	**100.0**	**12,209**	**12,209**	**/**	**0.17**
	Health insurance expenditures*	***100.0***	***6,465***	***6,465***	***100.0***	***5,558***	***5,558***	/	0.002
	Medication	99.4	1,394	1,394	99.5	1,277	1,277	/	0.12
	*including anti-dementia drugs*	*18.8*	*782*	*127*	*14.0*	*770*	*115*	*0.12*	*0.70*
	General practitioner	100.0	650	650	99.5	638	638	/	0.18
**Approach 2 two-part- model**	Medical specialist	88.2	675	595	79.1	447	363	<0.0001	<0.0001
	Hospital treatment	46.3	5,959	2,790	41.0	5,308	2,157	0.0005	0.01
	Non-physician services	28.3	839	225	26.5	677	190	0.25	0.03
	Medical aids	56.9	508	301	68.8	534	349	<0.0001	0.05
	Home health care	14.3	1,761	266	17.9	2,461	392	<0.0001	<0.0001
	Rehabilitation	5.2	3,307	170	4.6	3,459	164	0.81	0.21
	Long-term care services*	***59.3***	***9,027***	***5,935***	***68.3***	***10,267***	***6,467***	***0.065***	***<0.0001***

Per user data derive from one-step and two-step Generalized Linear Models with gender as the coefficient of interest.

Patient level data are means based on recycled predictions with gender as the coefficient of interest.

Significance at the patient level is estimated based on p1 and p2 in the two-part models and based on p2 in the one-step models.

p1 derives from the logistic model (approach 2, step 1) and p2 derives from the gamma model (approach 2, step 2 and approach 1).

* Results of model estimation; the addition of mean costs per category yields slightly different figures.

Within SHI, spending on home health care and medical aids was remarkably increased in female dementia patients, but male dementia patients incurred substantially higher expenditures regarding medical specialists, hospital treatment and non-physician services. Expenditures for rehabilitation, general practitioners, medication in general as well as for anti-dementia drugs in particular did not differ.

Within the control group, we also observed increased SHI expenditures in men and increased LTCI expenditures in women. Here, the comparably low LTCI expenditures failed to balance spending within both branches, leading to higher costs of formal care for men (results not shown).

### Age-dependent cost profiles

**Figure 2:**
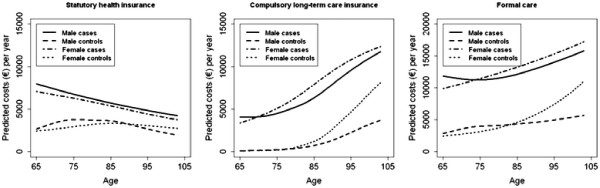
Age-dependent cost profiles for health care, long-term care and formal care.

Costs of formal care for dementia patients and control subjects increased with age. This effect was driven by rising LTCI spending, as SHI expenditures declined concurrently, as plotted in Figure 2.

Owing to increasing LTCI expenditures, costs of formal care for females exceeded costs for males at more advanced age, which affected cases about 10 years earlier than control subjects.

Within both genders, the differences in SHI expenditures, LTCI expenditures and costs of formal care between dementia patients and non-demented control subjects reduced slightly in older age.

In general, the impact of age on the three cost outcomes was highly significant (p < 0.0001) within all four subgroups. The only exceptions were borderline significance regarding costs of formal care in male dementia patients (p = 0.06) and insignificance regarding costs per LTCI user in male control subjects (p = 0.99).

These results remained stable when including proximity to death and comorbidity in the model.

To better understand the interactions between various covariates, we reverted to the extended model with interaction terms. As documented in Table 5, the age-dependent cost profiles of women progressed more steeply than those of men (impact of ‘age*female gender’ >1). Moreover, the age-dependent cost trend was less pronounced within the case group (impact of ‘age*dementia’ <1) but, apart from long-term care, the dementia diagnosis affected the curves of female patients more severely than those of male patients (impact of ‘female gender*dementia’ >1).

**Table 5:** Impact of various covariates on the costs of care

**Variable**	**Costs of formal care**		**Long-term care insurance**				**Health insurance**	
			**Probability of use**		**Costs per user**		**expenditures**	
	**Impact**	p-value*	**Impact**	p-value*	**Impact**	p-value*	**Impact**	p-value*
Dementia	2.84	<0.0001	16.85	<0.0001	1.87	<0.0001	1.71	<0.0001
Age~	1.19	<0.0001	3.64	0.003	1.10	<0.0001	1.05	<0.0001
Female gender	0.93	0.37	1.12	<0.0001	1.17	<0.0001	0.86	<0.0001
Age*female gender~	1.21	<0.0001	1.6	<0.0001	1.07	0.008	1.05	0.01
Age*dementia~	0.83	<0.0001	0.47	<0.0001	0.95	0.03	0.78	<0.0001
Female gender*dementia	1.12	0.0006	0.98	0.73	0.98	0.56	1.08	0.02

* p-values are derived from Generalized Linear Models (assuming a gamma distribution for costs and a binomial distribution for the probability of use).

~ Effects are reported per decade.

## Discussion

Adopting the perspective of the German social security system, our primary analysis revealed annual per capita costs for dementia patients of circa €12,300. This is in good agreement with previous national estimates based on patient-level data [7,8,15].

Dementia patients were approximately three times more expensive than non-demented control subjects. They incurred excess costs of around €8,300, about two-thirds of which occurred in the long-term care sector.

Compared with the BARMER GEK report (SHI: circa + €3,700; LTCI circa + €6,100) [15], we calculated the additional burden more conservatively, which might be explained by the older age structure of our sample combined with our finding that excess expenditures decline with older age. The figures from the AgeCoDe study (medical care: circa + €3,000; professional long-term care: circa + €9,400) [14] include out-of-pocket payments, rendering a direct comparison unfeasible.

Given a female majority among dementia patients [2,16], the women’s cost profile influences the cost structure of the entire population. Within the general older population, previous research observed significantly less spending on ‘acute medical services’ combined with significantly higher spending on ‘durable supportive services’ in females [24,25]. The latter is probably because older women are more likely to live alone than men of the same age and might thus have less access to informal care: older men perhaps still live with a (slightly younger) spouse who can act as an informal caregiver, whereas older women are more often widowed. Owing to the longer life expectancy of females, the living situation described is especially pronounced among the oldest of the old. Moreover, women were found to develop a comparable level of disability about 10 years earlier than men [25], which seems to be a further aspect leading to their increased usage of formal care services. Our secondary analysis validated this gender-specific cost compilation for individuals with dementia.

The cost profiles of the tertiary analysis revealed a continuous increase in LTCI spending accompanied by decreasing SHI expenditures in older age. The increase in LTCI spending overcompensates for the decline in SHI spending by a long way; thus, it seems obvious that demographic ageing affects the organization of long-term care services more seriously than the organization of health care services.

An interesting subsidiary finding of the tertiary analysis was the steadily declining SHI profile of dementia patients, which contradicted the expected inverse u-shape [26]. This picture might reflect a reduced ability of individuals with dementia to communicate their health care needs adequately [27] or even denote that expensive or interactive treatment strategies are not considered worthwhile in this population.

We are aware that our results need to be interpreted cautiously because of some methodological restrictions.

We did not adjust for comorbidity because we assumed a direct connection between dementia and the occurrence of several comorbid conditions and their respective treatment costs [11,28,29]. Including comorbidity as an additional confounder might have covered this specific impact of dementia on existing comorbid conditions. This decision seems not to result in remarkable bias as the comorbidity-adjusted sensitivity analysis yielded comparable results.

We also disregarded institutionalization within our regression models to avoid overadjustment as a dementia diagnosis is a strong predictor for nursing home placement [30-32]. The overall care strategies probably differ between community setting and institutional setting, which implies a setting-specific compilation of services.

Moreover, claims data as the single source of information imply specific advantages and disadvantages [33].

The accuracy of diagnoses remains uncertain, and the agreement of different data sources is insufficient regarding dementia disorders [34-37]. Requiring multiple dementia indicators enhances the validity of case group assignment but also selects more severe cases, whereas relying on one single indication implies a higher risk of false-positive classification. We consider our strategy to distinguish assured and potential dementia patients as a practicable solution, and suppose the ‘true’ excess costs of dementia care to lie in between the results of both analyses (circa €8,300 vs. circa €7,400). However, our approach regarded a minimum of three anti-dementia drug prescriptions within four consecutive quarters as on a par with a minimum of three dementia diagnoses in the corresponding period. By this method, we included 149 individuals (1.6%) who never received a dementia diagnosis despite it might have been more precise to assume mild cognitive impairment instead of manifest dementia in these cases. Given the low percentage, we desisted from excluding the individuals concerned.

Moreover, claims data lack information on disease severity. Thus, the impact of disease progression on costs [38] could not be accounted for despite the cost impact of disease severity is well documented especially in the field of long-term care [6,14,39]. Educational level, economic situation and family status are also only traceable rudimentarily from claims data, but the association between these variables and service utilization is broadly accepted. For example, living alone is a predictor for long-term care expenditures, because single people are presumed to have less access to informal support and therefore assumed to rely to a larger extent on professional help [40].

On the other hand, relying on claims data prevents the disregarding of considerable parts of the effective patient clientele. Observational studies and clinical trials based on primary information often do not address frail and institutionalized individuals. In contrast, claims data account for all insurants irrespective of health status and living environment and are thus less selective.

To ensure sound decision making, unambiguous economic information for the payer is paramount. Cost information from claims data are basically reliable, because they report the de facto spending on a broad range of medical and non-medical services. Thus, defining unit costs and extrapolating self-reported data becomes dispensable. The accuracy of such estimates is unclear, because assuming the recall period to be representative of the entire observation period seems questionable, and self-reported service utilization may be impaired by recall bias [41].

Regarding the future organization of dementia care, it must not be forgotten that, despite not being accounted for within a payer perspective, informal care is the crucial expense factor [14,39,42], especially in the community setting. The number of potential family caregivers will decrease because of changing living arrangements, increasing women in the labour force and decreasing birth rates in industrialized countries. Thus, a shift from informal towards formal care and a rising economic burden for social security systems can be expected all round the world. Our data provide information on the age- and gender-specific health and long-term care needs of individuals with dementia. This knowledge is a suitable starting point for developing targeted programmes to manage future care for this clientele efficiently.

## Conclusions

Our data suggest a special need to regard female dementia patients as a crucial target group. Owing to longer life expectancy, women face an increased risk of developing dementia. Moreover, females rely earlier and to a larger extent on professional long-term care than males. Dementia leads in turn to a comparatively early dependency on long-term care services. These services are the crucial expense factor in dementia care, and the corresponding expenditures rise steeply with age. Altogether, innovative strategies fostering community-based long-term care for females with dementia can influence the key driver of the financial burden resulting from dementia disorders. This would help to sustain a socially acceptable financing of comprehensive dementia care within the social security system.

## Competing interests

For LS, PMe, RL, RH (Helmholtz Zentrum München), and EG (University Hospital Erlangen - Clinic for Psychiatry and Psychotherapy) their institution received support from the funding organisations for the submitted work. SW (AOK Bavaria), HM (Eisai GmbH) and PMa (Pfizer Deutschland GmbH), are employees of one of the funding organisations and received a regular salary while contributing to the work; RL and EG received a honorarium from one of the funding organizations for presenting data or participating in an advisory board.

## Authors’ contributions

LS analysed the data and wrote the manuscript. PMe developed the statistical models and revised the statistics section of the manuscript. Together with RH, both decided on study design and the research questions to be answered. RL, SW, HM, PMa and EG advised on health care system-related issues and were involved in the constitution of the final study design. All authors contributed to the manuscript and approved the final version of the manuscript. RH is guarantor.

## Pre-publication history

The pre-publication history for this paper can be accessed here:

http://www.biomedcentral.com/1472-6963/12/165/prepub
